# Analysis of Received Signal Strength Quantization in Fingerprinting Localization

**DOI:** 10.3390/s20113203

**Published:** 2020-06-04

**Authors:** Syed Khandker, Joaquín Torres-Sospedra, Tapani Ristaniemi

**Affiliations:** 1 Faculty of Information Technology, University of Jyväskylä, Mattilanniemi 2, 40014 Jyväskylä, Finland; tapani.ristaniemi@jyu.fi; 2 Institute of New Imaging Technologies, Universitat Jaume I, Av. Vicente Sos Baynat s/n, 12071 Castellón de la Plana, Spain; jtorres@uji.es or; 3 UBIK Geospatial Solutions, Av. Vicente Sos Baynat s/n, 12071 Castellón de la Plana, Spain

**Keywords:** fingerprinting, quantization, indoor positioning

## Abstract

In recent times, Received Signal Strength (RSS)-based Wi-Fi fingerprinting localization has become one of the most promising techniques for indoor localization. The primary aim of RSS is to check the quality of the signal to determine the coverage and the quality of service. Therefore, fine-resolution RSS is needed, which is generally expressed by 1-dBm granularity. However, we found that, for fingerprinting localization, fine-granular RSS is unnecessary. A coarse-granular RSS can yield the same positioning accuracy. In this paper, we propose quantization for only the effective portion of the signal strength for fingerprinting localization. We found that, if a quantized RSS fingerprint can carry the major characteristics of a radio environment, it is sufficient for localization. Five publicly open fingerprinting databases with four different quantization strategies were used to evaluate the study. The proposed method can help to simplify the hardware configuration, enhance security, and save approximately 40–60% storage space and data traffic.

## 1. Introduction and Motivation

Positioning is an important part of our daily life. Essential services, e.g., navigation, health care, personnel management, and emergency rescue, require localization information to work effectively. The predominant localization technology (e.g., GPS and Galileo) using satellite signals has solved most of the outdoor positioning-related problems. However, the satellite-based positioning in indoor is severely degraded due to the blockage of the satellite signals by the obstacles. Therefore, alternative indoor localization technologies have been recently developed using optic [1], ultra-sound [2], dead reckoning [3], ultra-wideband [4], RFID [5], visible Light [6], and Bluetooth technology [7]. Most of them are based on communications technologies and require the deployment of additional hardware components in the environment to work effectively. Dead reckoning is only able to provide relative positioning (displacement from the origin) and needs a secondary technology to support absolute positioning and reduce the error drift. However, Wi-Fi fingerprinting localization has become a very promising and competitive technical solution for indoor positioning systems for its cost-effective high precision performance [8] for either smartphone [9,10] or autonomous vehicle [11,12] applications.

Wi-Fi fingerprinting localization is a technique that uses RSS measurement to perform localization tasks. RSS is easy to obtain with the current Wi-Fi interfaces without requiring any additional hardware. Moreover, unlike Channel State Information (CSI)-based techniques [13], it is not restricted to a particular LAN card and operating system. Therefore, RSS-based positioning has attracted considerable attention from researchers [14,15,16]. RSS is a measurement of the strength of a radio signal that performed at the receiver end. In a wireless network, RSS from different Access Points (AP) creates a unique pattern for a geographical area, which is called Wi-Fi fingerprint. It is assumed that a fingerprint from a recording point remains unique for that location. Therefore, fingerprints can be used to retrieve the location. Generally, the RSS values are expressed in dBm unit with 1-dBm granularity. The main aim of RSS is to check the signal quality for evaluating the coverage and the quality of service; therefore, fine-granular RSS is needed. However, in fingerprinting positioning along with RSS values, reference AP labels are also recorded. Since a fingerprint has many reference AP, instead of fine-granular RSS, coarse-granular RSS could be able to provide the same positioning accuracy. Moreover, storing, exchanging, and processing raw RSS value can occupy a considerable amount of memory [17,18], as well as be a potential threat to user’s location privacy [19,20]. In this case, a quantization method can be useful.

Quantization is the process of mapping a set of values to a particular value. It is inherent, for instance, in analog-to-digital converters and it has been widely studied in different positioning technologies [18,21,22,23,24,25]. In Wi-Fi fingerprinting, at the receiver end, a sensor captures the energy of a signal transmitted by the AP. This signal strength is a continuous number and fluctuates over time. For making sense out of it and for different kinds of signal processing, e.g., quality checking, ranging, and positioning, the signal power needs to be divided and expressed by a set of finite numbers, which is called quantization. The output (*l*) of a quantization depends on the used bit number. If *n* bits are used, the total output would be

(1) $$l=2^{n}$$

**Figure 1:**
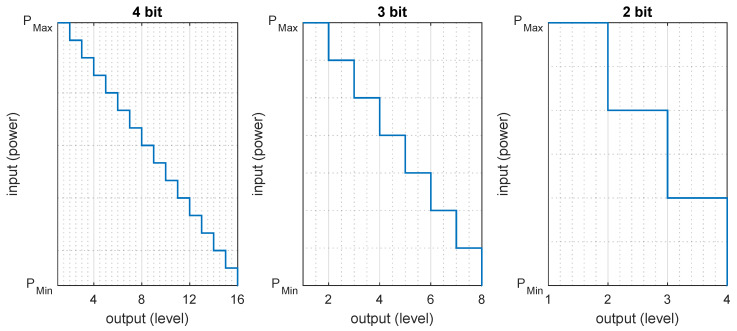
RSS quantization concept.

Figure 1 shows an example of linear quantization.

$P_{max}$ and $P_{min}$ are the maximum and minimum power of a signal. In Figure 1, we can see each quantized level shares a range of input power. Typically, the strength of a Wi-Fi signal is in between 1 and $10^{-10}$ mW. Through the quantization process, a large range of $P_{max}$ and $P_{min}$ can be scale down to a smaller set of numbers, where the granularity of output level depends on the used bit number. Figure 1 shows that more bits lead to finer output levels, while fewer bits lead to coarser output levels. The IEEE 802.11-2012 standard recommends to quantized the received signal power in the range of 0 to ≤ 8 bits, where the output levels are called Received Signal Strength Indicator (RSSI) [26]. However, quantization is implementation-dependent and done in a proprietary manner by different manufacturers. $P_{max}$, $P_{min}$, and applied bit number vary from manufacturer to manufacturer, which may contribute to the device heterogeneity problem, along with some other heterogeneity contributors such as the design of the antenna, and antenna orientation. Moreover, some manufacturers express the quantized value of receives power (e.g., 0,1,2,…,N) where some other provides RSS directly in dBm unit (e.g., −30 or −40) [27]. This makes a great confusion between RSS and RSSI. However, solving this confusion is out of the aim of this paper. More information regarding the relationship between RSS and RSSI can be found in [26,27,28].

Generally, in dBm unit, RSS values are in the range of 0 to −100 dBm, and expressed by 1-dBm granularity [29]. To express these 101 levels, at least 7 bits are needed. Some studies showed that, instead of this fine-granular RSS, coarse-granular or fewer-bit quantized RSS could provide the same positioning accuracy [17,18,30]. However, most of the simulation-based previous studies investigated the quantization effect on Wireless Sensor Networks (WSN) or Distributed Sensor Networks (DSN). RSS quantization in the Wi-Fi network focusing the fingerprinting positioning purpose has not been studied enough. Why coarse-granular RSS or reduced-bit quantization can provide the same amount of positioning accuracy is not investigated yet. In the real scenario, vast numbers of AP, different mobile devices, and obstacles can have a great impact on quantization. Lack of thorough investigation of RSS quantization on real data motivated us to conduct this research. The main contributions of this study are followings:

- We thoroughly investigated the usefulness of reduction bit quantization considering why it can perform the same positioning accuracy as that of traditional RSS.
- Reduction bit quantization also reduces the data size. We investigated how much disk space and network traffic can be saved if reduced-bit quantization were used.
- We analyzed the RSS quantization effect on five different publicly open fingerprinting databases. Our findings are coherent and reflect all the real-life scenarios.

The remaining of this paper is organized as follows. Section 2 introduces a review of the works related the quantization. Section 3 describes the fingerprinting databases and explains the method. Section 4 is devoted to the different kinds of experiments and results. In Section 5, we discuss the findings and observations. Section 6 presents the conclusions and draws the research lines for further work.

## 2. Related Work

Since the primary purpose of RSS is not localization, only a few works have been done on the technical aspect of RSS (i.e., quantization) focusing on the localization, especially in the Wi-Fi network. This section reviews the existing works that address different aspects of signal strength quantization.

Bahl and Padmanabhan introduced RADAR, the first indoor positioning system based on *k*-NN for Wi-Fi fingerprinting in 2000 [31]. Although some *k*-NN variants [32,33,34,35] and other advanced methods, e.g., probabilistic models [36,37] or SVM-based [38], have also been applied with fingerprinting, we focus on the simple *k*-NN model as it is still widely used nowadays [39,40,41,42,43].

Jarvinen et al. [44] proposed RSS quantization to secure two-party computation (STPC) to preserve the privacy in fingerprint-based positioning. Their proposal decreases the overhead of STPC evaluation. They implemented an approximately 500 times faster privacy-preserving indoor localization protocol using quantized RSS. They observed that a 50 % reduction on quantized bits has around 5% impact on the accuracy of localization.

Nguyen et al. [45] observed that, in a WSN at the outdoor environment, the quantized scheme performed more stably and had smaller errors compared to the indoor space; this is because the wireless signals in the indoor environment suffered from multi-path and reflection effects from walls and obstacles. Both RSS and angle of arrival measurement were used in their study. They mentioned that, after a certain number of quantization levels, positioning accuracy does not improve.

Ababneh [46] mentioned that in a distributed sensor network (DNS) the localization error becomes highly dependent on the total number of bits and the pattern of the distribution of the sensors, especially when the number of bits allowed is relatively small. He proposed two novel low-complexity bit allocation algorithms to solve optimal computational complexity. The simulation results show a significant reduction in computational complexity.

Richter et al. [17] studied the effects of the RSS quantization, focusing privacy-preserving. According to them, quantized RSS is beneficial to create ciphertext for encrypting purpose. Therefore, quantization can be an excellent tool for privacy preservation. Their non-uniform 4-bit quantization yields the same positioning accuracy as that of the unquantized system.

Nui et al. [47] performed a comparative study on location estimator in WSNs, based on quantized data, and compared it with localization approaches using analog data. They suggested that, to reduce communication costs, and sensor power consumption, local sensors should send quantized measurements to the fusion center. The simulation experiment showed that the positioning precision loss due to quantization reduces as the number of bits increases, which is almost negligible when 6 bits are used.

Li et al. [48] proposed quantized received signal strength-based target localization method to save energy and communication bandwidth in WSN. Their proposed algorithm, firstly, combines the particle swarm optimization (PSO) with their proposed formula to generate a fixed set of thresholds and then uses those to quantize the measured RSS. Based on the obtained quantized RSS data, PSO and optimized algorithms are used for accurate and efficient positioning.

Gao et al. [30] showed that, in fingerprinting positioning, the full resolution RSSI measurements are unnecessary. According to them, raw RSSI are noisy, exhibit a high degree of variability, and occupy a massive amount of memory. In their study, through a non-uniform quantization process, it was possible to reduce the RSSI data volume by approximately 72% without compromising the localization accuracy, while the positioning accuracy was around 2 m.

Torres-Sospedra and Moreira [49] analyzed the sources of very large positioning errors. They identified that the representation of the received signal strength as an integer value, which can be seen as a first quantization added to all RSSI measurements, added some uncertainty to the reference fingerprints, especially if the distance between the AP and the receiver is moderate or high. Depending on the AP distribution, which in most scenarios were deployed for communication purposes, and the noise level in the RSSI measurements, there can be some large areas in the operational environment where the collected fingerprints are similar. Thus, the fingerprint methods cannot properly operate or provide accurate position estimations on them.

Mizmizi and Reggiani [18] showed that the computational complexity could be limited by adapting the RSSI quantization. Their simulation of WSN contained many beacons over a limited squared area on a single floor with a two-dimensional coordinate system. They observed that there is an optimal quantization level number, over which positioning accuracy cannot be improved without increasing the number of beacons. However, if the quality of the RSSI measurements increases, then it is possible to reduce the number of quantization levels at the same performance. The exact meaning of the “quality of the RSSI” was not mentioned.

Shi et al. [50] studied the relationship among quantization level, network configuration parameters, and the lower bound of the positioning error based on the quantized RSSI in WSN. Their simulation suggests that localization error variance cannot be improved by only increasing the quantization level after a particular value; this is due to the noise in the RSSI readings. When the quantized RSSI value interval is comparable to the noise, increasing the quantization level becomes less useful.

Krishnamachari [51] showed a positioning performance analysis by using Cramér–Rao bound (CRB). He mentioned that 3 bits of RSS quantization in WSN suffices to give a lower bound that is very close to best possible.

Patwari and Hero [52] used the Cramer–Rao Bound (CRB) to compare the minimal attainable variances of unbiased sensor location estimators in WSN. They considered that RSS measurements are always going to be quantized while an analog-to-digital converter converts it. If there are many levels, then the effect of the quantization is minimal. According to them, 3 bits of quantization are sufficient for an RSS-based positioning system.

Most of the previous works were done in WSN or DSN. According to our best knowledge, only two studies were done on RSS quantization in the Wi-Fi network focusing on user’s privacy preservation [17,44]. In general, the RSS quantization effect in the Wi-Fi network has not been studied enough. Moreover, simulation results of the previous studies may not reflect all the aspects of a real-world scenario. This study focused on a step-by-step investigation of the quantization process on five different publicly open databases by four different quantization strategies. Furthermore, we considered applying quantization on the absolute signal energy level, which is a novel contribution to the Wi-Fi fingerprinting localization. Finally, the quantization of RSS measurements in fingerprinting has usually been applied to reduce the radio map size and optimize the computation of fingerprint-based methods. However, quantization only partially reduces the complexity of fingerprinting as the number of APs and reference fingerprints are not altered. Other relevant works that have focused on reducing the radio map include a focus on removing useless APs from the radio map [53], applying unsupervised learning to cluster/split the radio map [33,38,54,55], using radio-signal propagation knowledge to filter fingerprints out the radio-map [34,56,57,58], combining clustering with RSSI-based rules [59], and, even, reducing on-the-fly the reference samples and APs of the radio map [60]. In this study, we focused on the nominal case where no other optimizations have been performed. This ensures that the study reflects the benefits of quantization without the interference of other approaches, such as advanced fingerprint models or optimization rules.

## 3. Materials and Methods

In this section, we describe fingerprinting databases and the RSS quantization process.

### 3.1. Database Description

Five publicly open fingerprinting databases were used in this research. The first one is from the University of Jaume I, Spain. The author presented their work at the 2014 International Conference on Indoor Positioning and Indoor Navigation conference; the name of this database is UJIIndoorLoc [61]. The second one is from the Tampere University of Technology (TUT) Finland; we named it as TUT database [62]. The third database was collected by the research group of the University of Minho, Portugal, and is called Minho database [63]. The name of the next database is Mannheim. It was collected by the research group from the University of Mannheim, Germany [64]. The final database was collected from a library building of the University of Jaume I, which is different from the first database; we call it the Library database [65]. Short descriptions and comparison of these databases are discussed below.

#### 3.1.1. UJIIndoorLoc Database

This multi-building, multi-floor localization database is the first publicly available Wi-Fi fingerprinting database created by the researchers from the University of Jaume I, Spain. Twenty users and 25 devices were deployed to collect the fingerprinting data from 3 buildings with 4 or 5 floors depending on the building. Two Android applications were used to create the database. In total, this database contains 21,049 samples. Apart from the RSS and location information, features such as BuildingID, FloorID, UserID, and timestamp are given for each sample. The location points were uniformly distributed to the users with the restriction that any reference point should be covered by, at least, two users. The longitude and latitude coordinates are given in meters with UTM from WGS84.

#### 3.1.2. TUT Database

Fingerprints from the TUT university building were collected by a research group from that university from January 2017 to August 2017. The total area of that building is approximately 22,570 m${}^{2}$ containing five floors. In total, 991 AP were recorded during the collection period. Out of 4648 collected fingerprints, 15% were used for the training and 85% were used for the testing. All fingerprints contained local coordinate values, e.g., (x, y, z) = (41.45, 14.71, 3.7), not the GPS one. Twenty-one Android devices having an identical application were used during the crowd-sourced data collection process. According to the TUT research group, approximately 10 m of mean 3D positioning accuracy was achieved through different positioning methods.

#### 3.1.3. Minho Database

The research group from the University of Minho, Portugal collected the third database of this study from a university building that resembles an industrial floor plant, with a total area of around $1000\;m^{2}$. The data were collected in July 2017. The data collection setup was based on a Raspberry Pi 3 Model B with its internal Wi-Fi interface, and four additional USB Wi-Fi interfaces (Edimax EW-7811un). The database contains five sets of files, one for each one of the Wi-Fi interfaces. Since there is no significant impact of change in the Wi-Fi interface in our study, we used the data from the fifth interface in our work. In total, 5783 fingerprints were collected, among which 4973 were labeled as training fingerprints, and the rest were used as test fingerprints. The RSS from 11 different APs were recorded in each fingerprint.

#### 3.1.4. Mannheim Database

A research group from the University of Mannheim, Germany, collected this database. The primary purpose of this database is to create a digital compass. Therefore, apart from the RSS and location, angular information was recorded by a USB powered digital compass. Samples were collected from the hallway of an office building. For each training and testing point, samples were collected 20 and 100 times, respectively. However, to speed up the experiment, we randomly selected ten samples per testing points. We had 14,300 training samples and 5060 testing samples. Local X,Y coordinate was used to define the location of the fingerprints.

#### 3.1.5. Library Database

The UJI research group has been collecting the Wi-Fi measurement from their university library premises for the last 25 months, which has $308\;m^{2}$ of footprint. The fingerprints were recorded from the bookshelves area of the third and fifth floors of the library. These two floors are adjoining floors despite their numbering. Wi-Fi signal could travel from one story to another through stairs, but no line-of-sight path is available. That data have been recording for the last 25 months for the same place. We used the latest data (25th month) in this study. The database contains 576 training fingerprints and 3120 testing fingerprints. As with the TUT database, they also used a local coordinate system. In total, 620 AP were heard during the collection time. A Samsung Galaxy S3 smartphone was used to collect the fingerprint data with dedicated software. The authors reported the mean 2D positioning accuracy of 2.34 m.

### 3.2. Facts in the Databases

Facts regarding fingerprinting positioning in all theses database are summarized in Table 1.

Positioning performance based on traditional RSS was calculated using the *k*-NN algorithm and Euclidean distance. To keep similarity with previous works, *k* = 1 was used in UJIIndoorLoc, TUT, and Mannheim; and *k* = 3 was used in Minho and Library. Positioning accuracy for the TUT database was based on 3D positioning; for the other databases, it was 2D positioning. Minho and Mannheim have no floor information; therefore, floor detection is Not Applicable (NA) in these two.

### 3.3. Method

We briefly state the quantization process in the Introduction. In this subsection, the proposed quantization method is explained in detail.

#### 3.3.1. Quantization

The Wi-Fi signal is an electromagnetic wave whose intensity is attenuated as it propagates through space. When that wave reaches the antenna of a sensor, the sensor captures the energy from the signal. This energy expresses the strength of that signal. Despite the signal strength being continuous, it needs to be expressed to a countable finite number of elements to make that sensible and valuable for a different kind of signal processing, e.g., checking the quality of the signal, ranging, and positioning. To express the continuous value of the received signal to finite numbers, it generally goes through a process called quantization. If $P_{i}$ is the absolute received power (in mW), using an *S*-level quantization, we obtain

(2) $$Q\left(P_{i}\right)=\left\{\begin{array}{cc} 0, & L_{0}\leq P_{i}<L_{1}\\ 1, & L_{1}\leq P_{i}<L_{2}\\ . & .\\ . & .\\ . & .\\ S-1, & L_{S-1}\leq P_{i}<L_{S} \end{array}\right.$$

**Figure 2:**
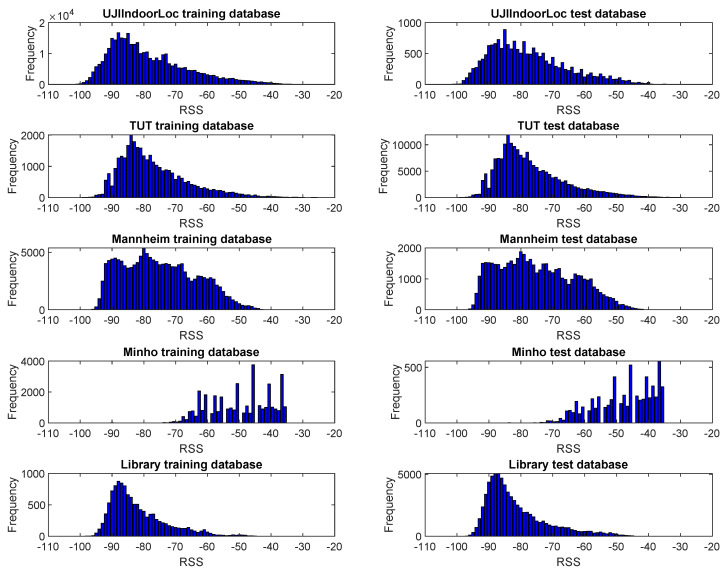
RSS distribution in the databases.

Here, $L_{0},\ldots ,L_{S}$ defines the range of the quantization levels, where $L_{0}=\infty$ mW and $L_{s}=-\infty$ mW. The quantized power is $Q(P_{i})=0,1,\ldots \ldots ,S-1$. If we look at the distribution of RSS in all the databases in Figure 2, we can see that the spreading of RSS is between −30 and −100 dBm. However, there could be some negligible amount of RSS reading beyond this range. It is common practice to use very weak signal value, e.g., −103 dBm for the non-heard AP in the fingerprints, to maintain the same length for all the fingerprints. Thus, we can see RSS values in the scale of −30 to −103 dBm are mostly used in fingerprinting positioning. RSS beyond this range will have no or negligible impact in fingerprinting localization. Therefore, we considered quantizing the RSS only from −30 to −103 dBm. The databases provide RSS in dBm unit. The following equations can convert RSS value from dBm to mW and vice versa.

(3) $$P_{\mathrm{mW}}=1\;\mathrm{mW}\times 10^{\frac{P_{\mathrm{dBm}}}{10}}$$

(4) $$P_{\mathrm{dBm}}=10\times \log_{10}\left(P_{\mathrm{mW}}/1\;\mathrm{mW}\right)$$

Since quantization happens at the absolute signal energy level, at first, we converted all RSS from dBm unit to mW unit using Equation (3) and then applied quantization. However, we found that, instead of mW unit, if quantization is applied on dBm unit, it provides the same results because they are just the same value represented by two different units.

#### 3.3.2. Quantizer

Figure 2 shows that the usable RSS range for fingerprinting localization is −30 to −103 dBm, which is equivalent to $10^{-3}$ to $10^{-10.3}$ mW. Thus, we can set maximum energy index, $E_{max}=-3$, and minimum energy index, $E_{min}=-10.3$. We proposed four different formulas ($f_{1},f_{2},f_{3},f_{4}$) for seven different bit numbers (n) where n=2,3,4,5,6,7,8 and $i=1,\ldots ,2^{n}$ to divide the signal energy for each quantization level. The first formula results a linear quantization; depending on applied bit number, each level shares an equal amount of energy index.

(5) $$f_{1}=\left(E_{max}-E_{min}\right)/\left(2^{n}-1\right)$$

In the next three formulas, we applied non-linear quantization to reflect the nature of signal propagation. From the maximum energy index, in each quantized level, the energy index is reduced as follows

(6) $$f_{2(i)}=\left(E_{max}-E_{min}\right)\times \frac{\sum_{i=1}^{i_{th}}\sqrt{2^{i-1}}}{\sum_{i=1}^{2^{n}}\sqrt{2^{i-1}}}$$

(7) $$f_{3(i)}=\left(E_{max}-E_{min}\right)\times \frac{\sum_{i=1}^{i_{th}}\sqrt[{3}]{2^{i-1}}}{\sum_{i=1}^{2^{n}}\sqrt[{3}]{2^{i-1}}}$$

(8) $$f_{4(i)}=\left(E_{max}-E_{min}\right)\times \frac{\sum_{i=1}^{i_{th}}log\left(2^{i-1}\right)}{\sum_{i=1}^{2^{n}}log\left(2^{i-1}\right)}$$

**Figure 3:**
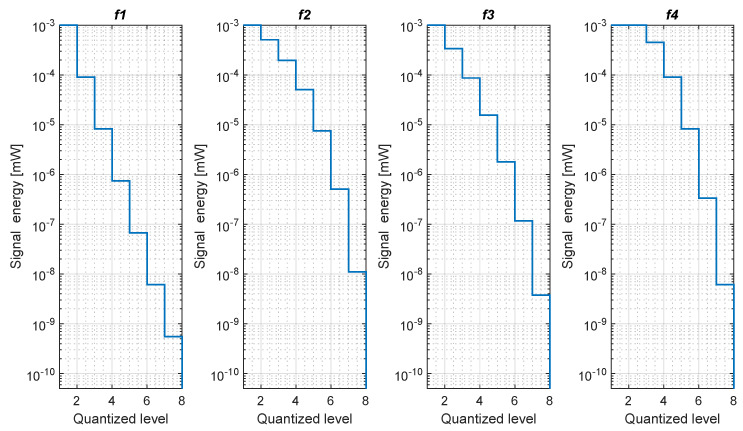
Three-bit quantization using proposed formulas.

**Figure 4:**
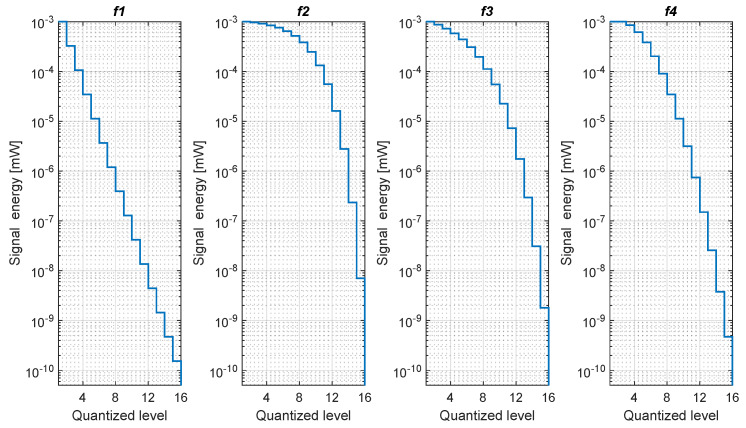
Four-bit quantization using proposed formulas.

Figure 3 and Figure 4 show the 3- and 4-bit quantizers, respectively.

#### 3.3.3. Reason for the Same Positioning Performance by the Traditional and Quantized RSS Fingerprint

**Figure 5:**
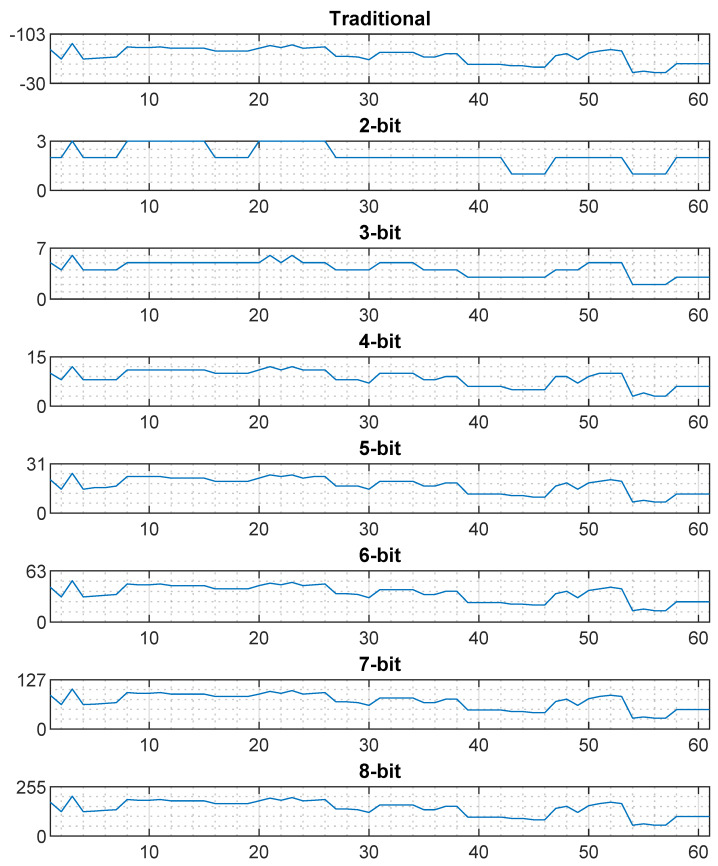
Traditional vs. quantized RSS fingerprint.

The proposed quantization scales down the traditional RSS values. To check the characteristic of an entire fingerprint before and after quantization, we randomly choose a sample from the TUT database. This example is based on linear quantization. This particular sample received signals from 61 APs. Figure 5 shows the fingerprints in traditional and quantized RSS representation, where the *X*-axis shows the reference AP and the *Y*-axis shows the RSS values. The 2-bit quantization has maximum capacity of four output levels, where each level represent s$\left(-30-\left(-103\right)\right)/\left(2^{2}-1\right)=24.33$ dBm equivalent signal power. Thus, 24.33 dBm traditional RSS is mapped to each quantized level, where scaling ratio is 24.33:1; similarly for 3 to 5 bits, the scaling ratio is 10.42:1, 4.86:1, and 2.35:1, respectively. If the scaling ratio gets closer, more characteristics of the original fingerprint appear. Figure 5 shows that 2-bit quantization misses many characteristics of the traditional one, while the situation improves at 3-bit quantization. The 4-bit quantization seems to have most of the characteristics of the original fingerprint at a lower magnitude.

**Figure 6:**
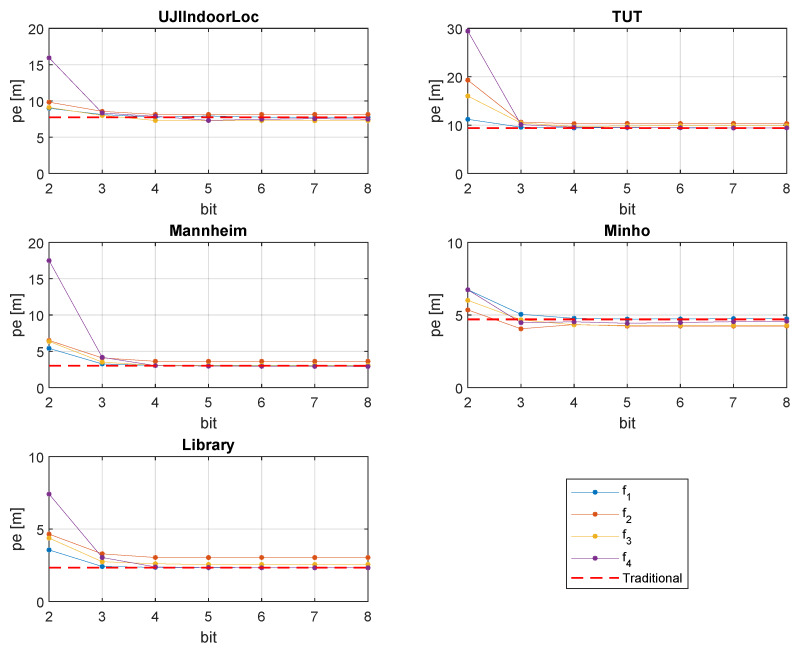
Positioning performance.

For the same amount of attenuation, a strong Wi-Fi signal travels less distance than that of a weak signal. The 2-bit quantization’s granularity is 24.33 dBm. According to the ITU-R indoor propagation model, for this amount of attenuation, a 2.4 GHz Wi-Fi signal can travel approximately 7 m when it is strong or more than 30 m when it is weak [66]. Here, we considered approximately −30 dBm as strong and −70 dBm as weak signal levels. Due to this high-granular RSS, depending on the composition of a fingerprint (e.g., number of AP and signal quality), there is a possibility of having identical quantized fingerprints for many different locations, which is a problem. That is why we can see a significant amount of positioning error for low-bit quantization in Figure 6. If the granularity is reduced, the problem mitigates. At 4-bit quantization, the granularity is only 4.86 dBm. This amount of attenuation can cover approximately 1 m and 6 m at the strong and weak states, respectively. Since the attenuation covers less distance, the possibility of having identical quantized fingerprint for the different locations also diminishes. Besides, the number of reference APs and a wide variety in signal level also helps a quantized fingerprint to be distinct from other quantized fingerprints. If each quantized RSS fingerprint is unique at their signal space, they will result in the same positioning accuracy as that of traditional RSS fingerprint. That is why 4-bit quantized fingerprint can achieve the same positioning accuracy as the traditional one. However, the situation may vary from sample to sample and environment to environment. Therefore, we carried a series of experiments on five different databases.

## 4. Experiment and Results

This section provides information regarding experiment and localization performance for different quantization setup.

### 4.1. Experiment Setup

Previous studies on these databases were mostly done based on popular Euclidean distance and *k*-NN algorithm [61,62,63,64,65,67]. To keep consistency with earlier studies, *k* = 1 was used in UJIIndoorLoc, TUT, and Mannheim. *k* = 3 was used in Minho and Library. If we change any parameters (in this case, *k* value), the experimental results would show not only the effect of proposed quantization but also a mixed impact of quantization and change of *k* value. However, our main target was to identify only the quantization effect. Therefore, we kept the k=1 in some databases, and k=3 in some other databases to keep similarity with the previous study. The distance between the ground truth and the calculated position (based on RSS) of a fingerprint is called positioning error (pe), which has been expressed in meters. Fingerprints in the TUT database have exact height information (not just the floor level number, but height in meters); therefore, we calculated 3D pe for the TUT database, For the other databases, it was 2D positioning. Our laboratory computer had an Intel Core i5-6600 processor with a clock speed of 3.3 GHz and 8 GB RAM, using Matlab 2018a programming software.

### 4.2. Positioning Performance

Figure 6 shows the quantization effect on fingerprinting positioning for all the databases. To compare the result with the traditional RSS representation, a red dashed line is drawn. We did not consider 1 bit because that results in a huge amount of pe. Two-bit quantization also shows a large pe. However, from 3-bit quantization, positioning accuracy dramatically improves. Dimension, environment, and the number of training and testing samples are quite different in all the databases. As a result, the localization performance slightly varies by different quantization formula. However, the 4-bit quantization results in almost the same positioning accuracy as that of traditional RSS. Beyond 4-bit quantization, positioning accuracy does not improve. Formulas $f_{1}$ and $f_{4}$ provide slightly better performance then that of formulas $f_{2}$ and $f_{3}$.

### 4.3. Floor Detection Performance

**Figure 7:**
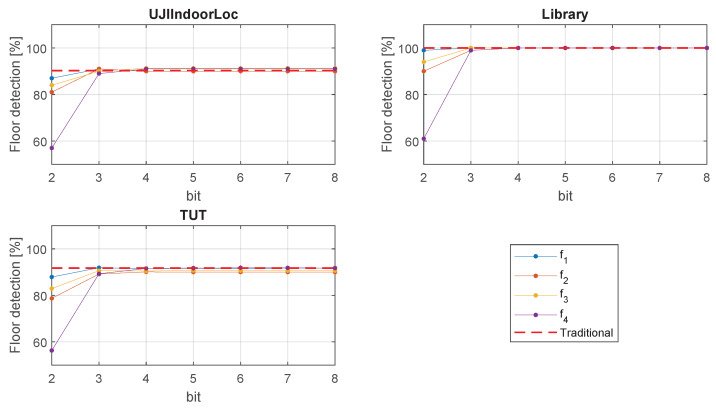
Floor detection performance.

We also checked the efficiency of our proposed method for successful floor detection. Figure 7 shows the successful floor detection rate in Library, TUT, and UJIIndoorLoc. The other databases had no floor related information. In Figure 7, we can see that, in most cases, the performance of 2-bit quantization is not satisfactory. However, from 3-bit quantization, the successful floor detection rate starts to get closer to that of traditional RSS results. Depending on the database, performance slightly varies by the different formulas. Formula $f_{1}$ is the best performer in all the databases.

## 5. Discussion

Our proposed method uses only the effective portion of RSS for fingerprinting positioning purposes. The result from the experiments shows that our proposed quantization method provides the same positioning performance as that of the traditional RSS representation by using only 4 bits. Since our proposed method uses only 4-bit quantization, it may bring the following advantages.

### 5.1. Simplification

Figure 2 shows that RSS from −30 to −103 dBm (or equivalent from $10^{-3}$ to $10^{-10.3}$ mW) is used in fingerprinting. Besides, a maximum 4-bit or 16-level quantization of that range is sufficient for the localization. Therefore, instead of a traditional 8-bit quantizer, a 4-bit quantizer can be used, which would simplify the hardware configuration and the sensing process.

### 5.2. Enhancing Security

Traditional RSS contains real signal strength information. Storing and exchanging this raw information could be a potential threat to the user’s location privacy. An adversary in between client device and positioning server may eavesdrop and get the information; later, through a radio signal propagation model, the position of the user could be determined. However, our proposed method does not use the real RSS values, but the mapped one according to a hidden quantization formula and bit number. Therefore it would be significantly harder for any adversary to get the real RSS, and subsequently the position.

### 5.3. Less Storage

**Figure 8:**
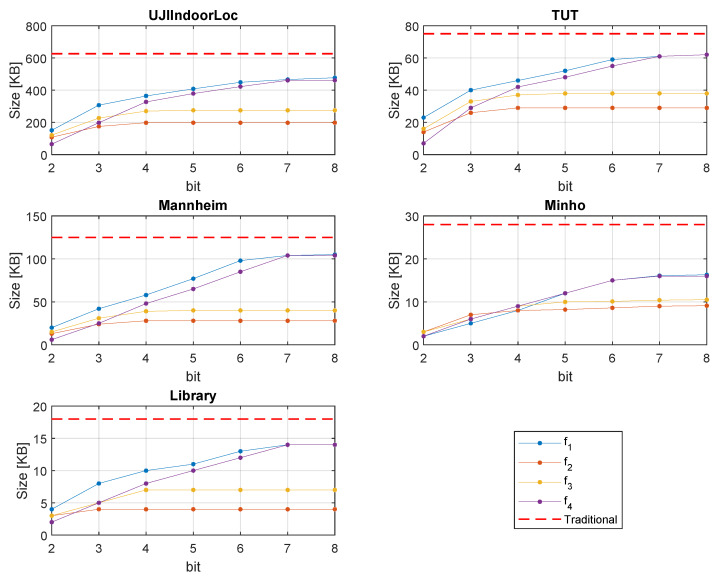
Training database storage size comparison.

The representation of traditional RSS and quantized RSS are different. Since we use fewer bits, less memory will be occupied. Figure 8 shows the comparison of the storage space of the training databases, which generally located at the positioning server side. The efficiency and quantization strategy of all the proposed formulas are not the same. As a result, different formulas result in different memory sizes. In Figure 3 and Figure 4 we can see $f_{2}$ and $f_{3}$ are biased to the strong part of the signal. Therefore, with the increase of bit number, the strong part of the signal gets more quantized, while a big chunk of the weak part of the signal is quantized to a single level.

As a result, disk size through $f_{2}$ and $f_{3}$ does not increase gradually. However, $f_{1}$ and $f_{4}$ quantize the signal linearly and show a gradual increase of memory size with the rise of bit number. In Figure 8, we can see that, compared to the traditional RSS, on average, 40–60% memory space in the training databases can be saved by 4-bit quantization.

### 5.4. Less Traffic

**Figure 9:**
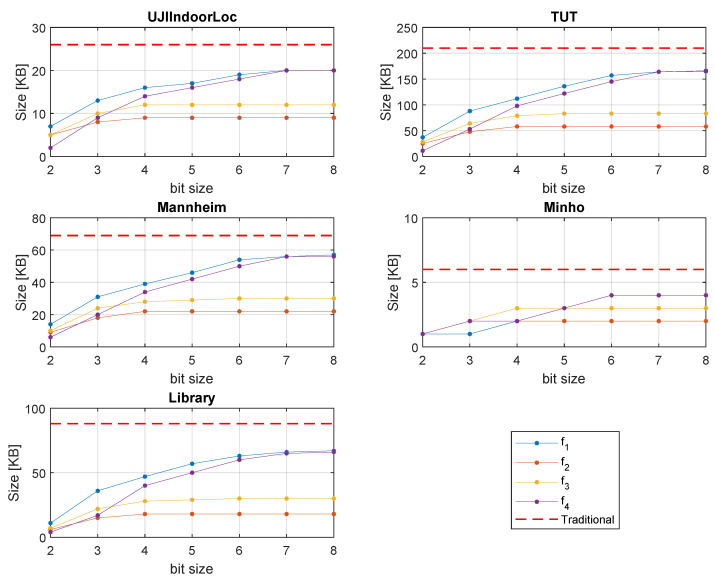
Test database storage size comparison.

Users having no positioning information would request the server by exchanging test/online fingerprint. Thus, the data need to be exchanged between the client device and the positioning server. Since our proposed method scales down the data size, compared to the traditional RSS, there is less traffic while exchanging the online fingerprint. Figure 9 shows the test data size. Since the default values of non-heard APs are handled at the server-side, we excluded the non-heard AP reading from all the test fingerprints. Figure 9 shows that, through the proposed method, there is an excellent opportunity to reduce the network traffic while exchanging the online fingerprint. Depending on the database and applied formula, compared to the traditional approach, on average, 40–60% network traffic reduction is possible by the proposed 4-bit quantization.

#### Observation

Based on the outcome of the experiments, we can draw the following observations:

- The effective range of RSS is from −30 to −103 dBm for fingerprinting localization. Any value beyond this range can be considered as noise.
- In Wi-Fi fingerprinting localization, 4-bit quantization is enough.
- To have good quality fingerprints, a well planned, organized Wi-Fi network is desirable.
- Sometimes, the number of APs per fingerprint in the crowd-sourced database is very high. RSS from the reliable or known AP should be used.
- Hot-spot or mobile AP can contribute a significant amount of noise. Data from those should be ignored.

## 6. Conclusions

In this study, we investigated the received signal strength quantization effect for localization in five different databases by four different formulas. The environment, data collection strategy, coverage area, number of training and testing fingerprints, number of floors, and number of APs were different from one database to another. Without having any correlation in all the databases, we found that 4-bit quantization can achieve the same localization accuracy as that of traditional RSS. Generally, the traditional RSS is quantized by 8 bits. However, most Wi-Fi chip manufacturers do not reveal their quantization strategy. In general, we can see the typical range of the RSS is between 0 dBm and −100 dBm that needs 101 different levels, which is in the range of at least 7 bits. In our study, we found that, for fingerprinting positioning purposes, 4-bit or 16-level quantization is sufficient. The proposed quantization method can help to simplify the hardware configuration, conceal the real RSS, save approximately 40–60% storage space, and data traffic. Since the quantization happens just after sensing the signal, we used the signal strength in the mW unit. However, we found that, instead of mW format, if quantization is done based on the dBm unit, that also provides the same localization accuracy. The performance of all the quantization formulas slightly varies. Nonetheless, linear quantization is a consistent performer. We achieved the performance limit of traditional RSS through 4-bit quantization.

One limitation of this study is that quantization was analyzed using *k*-NN as the main localization algorithm. Probably, quantization will have a similar impact on the results reported by the variants based on weighted *k*-NN. Therefore, as further work, we shall focus on how to reduce the computational load and increase localization accuracy through an efficient combination of RSS quantization, radio map clustering, AP filtering, and advanced fingerprint algorithms.

## Figures and Tables

**Table 1:** Facts in the databases.

Database	Coverage ($m^{2}$)	Number of Training Sample	Number of Testing Sample	Number of AP	Positioning (m) Accuracy	Floor Detection (%)
UJIIndoorLoc	108,703	19,936	1111	520	7.74	90.28
TUT	22,570	697	3951	991	9.39	91.75
Minho	1000	4973	810	11	4.7	NA
Mannheim	312	14,300	5060	28	3.01	NA
Library	308	576	3120	620	2.34	100
